# Larger Blood Pressure Reduction by Fixed-Dose Compared to Free Dose Combination Therapy of ACE Inhibitor and Calcium Antagonist in Hypertensive Patients

**Published:** 2017-07-01

**Authors:** Valeria Visco, Rosa Finelli, Antonietta Valeria Pascale, Rocco Giannotti, Davide Fabbricatore, Nicola Ragosa, Michele Ciccarelli, Guido Iaccarino

**Affiliations:** 1 Department of Medicine, Surgery and Dentistry, University of Salerno, Italy

**Keywords:** Hypertension, combination therapy, ACE Inhibitors, Calcium Antagonist, Blood pressure control

## Abstract

The introduction of fixed combination of ACEi+CCB (Fixed) has significantly increased patients compliance and adherence to therapy. At the moment, however, there are no data suggesting the better control of once-daily fixed (Fixed) over free doses in separate administrations combination therapy in hypertensives. In a population of 39 consecutive outpatient patients referred to the departmental Hypertension clinic of the University Hospital of Salerno Medical School with the first diagnosis of arterial hypertension, we tested the hypothesis that the Fixed achieve a better control of blood pressure than the Free combination. Patients were randomized to either strategy and after 3 months patients underwent a clinical assessment to evaluate the antihypertensive effect. The two groups, matched for anthropometric and clinical parameters, received Amlodipine (5–10 mg/daily) and Perindopril (5–10 mg/daily). Perindopril and Amlodipine doses did not significantly differ between the two groups. After 3 months BP control was improved in both groups and BP targets were similarly reached in both groups (SBP; Fixed: 61.54%; Free 69.23%; n.s. DPB; Fixed: 80.77%; Free 84.62%; n.s.). The reduction in systolic blood pressure was similar in both groups (Fixed:7.64±2.49%; Free: 7.81±4.00%, n.s.), while the reduction of diastolic blood pressure was greater in the Fixed group (Fixed: 14.22±2.03%; Free: 4.92±5.00%, p<0.05). Although both strategies are effective in reducing BP, the use of Fixed dose has an advantage in the reduction of BP. The present study does not allow to identify the mechanisms of this difference, which can be assumed to be due to the pharmacokinetics of the drugs administered in once-daily fixed combination.

## I. INTRODUCTION

Hypertension is a global public health problem and its treatment is primarily aimed to reduce associated cardiovascular morbidity and mortality.

Many observational studies show that hypertension control is still largely insufficient^1^ and recent studies have shown that only 20–30% of patients in drug treatment reaches the recommended pressure values in Europe^2^–^4^, emphasizing the importance of developing novel strategies for the management of this condition.

Blood pressure control involves changes in lifestyle, including caloric intake restriction, exercise and smoke cessation, but in most cases the final strategy is pharmacotherapy.

The pharmacological approach aims at reducing BP levels through an action on the peripheral resistance, cardiac output, or both factors. The choice for the initial therapy is from one of five classes of antihypertensive drugs, including diuretics (thiazides, chlorthalidone, and indapamide), beta blockers, calcium channel blockers (CCB), angiotensin-converting enzyme inhibitors (ACEi) and angiotensin II receptor antagonists (ARBs), either alone or in combination. Since there are no certain data to demonstrate the real superiority of a class of drugs over the others^5^–^7^, the choice of drugs should be individualized to each patient and may be influenced by the possibility of side effects, efficacy, safety, and by results of randomized controlled trials in specific populations of patients with arterial hypertension^8^.

Per ESH/ESC 2013 hypertension guidelines, regardless of the drug used, the monotherapy reduces the BP only in a limited number of hypertensive patients^9^. Therefore, the majority of patients requires the combination of at least two drugs to achieve BP control^9^. A recent meta-analysis of 42 studies has demonstrated that the combination therapy reduces the blood pressure values much more than the use of a single drug in double dose^10^. The synergistic effect of dual combination therapy provides not only the hypotensive activity but also a better prevention of therapy complications. The concurrent use of drugs with different mechanisms of action can offset the potential adverse effects of each compound. The combination of drugs of complementary classes increases effectiveness in reducing BP about 5 more than the simple increase in the dose of a drug^10^.

Adherence to treatment in the long term is necessary to BP control, and combination regimens can facilitate both the reduction of the number of drugs and the frequency of dosing required; in this regard, a recent study has found that adherence was inversely proportional to the number of prescribed drugs^11^.

Among the combination therapies which may be employed in treatment of BP, we must choose the most efficient combinations to reduce the global cardiovascular risk profile and increase safety and tolerability. The use of a strategy based on the combination of drugs which antagonize the renin-angiotensin system is able to significantly reduce the risk of major cardiovascular events^12^ and discontinuation of therapy^13^. The Accomplish study^14^ found a significant superiority of the ACEi associated with a CCB compared to the association ACEi/diuretic. The combination amlodipine-perindopril has been widely used in the ASCOT study, being more effective in lowering blood pressure (BP) and cardiovascular events than the combination of a beta-blocker with a thiazide^15^. Moreover, through their sympatholytic effects, ACEi attenuate the increase in heart rate that can occur during treatment with a dihydropyridine CCB. In addition, ACEi reduces the peripheral edema, which is a limiting side effect of calcium channel blockers^16^, so the ACEi+CCB combination is particularly recommended^9^. In this regard, the fixed combination ACEi/ARB + CCB appears particularly promising as it can significantly reduce BP, improve the cardiovascular outcome, prevent organ damage, improve adherence to therapy. The use of the combination of two antihypertensive drugs at fixed doses in a single tablet reduces the number of pills that must be taken daily, with a better compliance to therapy^17^. Single-pill combinations are now widely available because at low doses fixed dose combinations may have greater efficacy and better tolerability than monotherapy^18^.

Nevertheless, it is not clear whether the fixed dose therapy presents any advantage on BP control compared with free dose combination therapy. In this regard, the aim of our study was to assess whether the use of fixed-dose (ACEi+ CCB) produces better control of BP in hypertensive patients compared with the free dose.

## II. MATERIALS AND METHODS

### A. Study Population

Our study included 39 patients referred to the Hypertension Clinic of Salerno Medical School Hospital in Salerno, with the first diagnosis of arterial hypertension and in the absence of a previous treatment. At the time of enrollment visit, patients signed a consent to anonymous participation, in compliance with the regulations of good clinical practice and privacy. Study participants were 18–75 years old with essential hypertension (defined according to the ESH/ESC 2013 guidelines). Patients were excluded if they had secondary hypertension, malignant hypertension, CRF (chronic renal failure), oncological conditions or cirrhosis. Patients were also excluded if they had medical and surgical disorders that alter absorption, distribution, metabolism and excretion of drug treatment. The study protocol was approved by the competent University Hospital Ethical Committee.

### B. Study Design

Patients were randomized to either fixed dose or free dose combination therapy, with Perindopril (5 or 10 mg) and Amlodipine (5 or 10 mg) with a 2:1 randomization design based on a power analysis. Doses were decided according to anthropometric, clinical, biochemical and instrumental doses by experienced medical staff. The Fixed group received one single tablet containing Perindopril/Amlodipine at the appropriate dose. The Free group, received Perindopril and Amlodipine in separate tablets at the appropriate dose. Groups were matched for age, sex, BMI, systolic BP (SBP) and diastolic BP (DBP). At baseline and at follow-up we evaluated clinical (weight, height, BMI, heart rate, BP) and biochemical parameters (blood glucose, serum cholesterol, LDL, HDL, triglycerides, blood urea nitrogen, creatinine, creatinine clearance), as well as Electrocardiogram (ECG) and cardiac ultrasound.

### C. Clinical parameters

In accordance with the ESH guidelines^9^, BP assessment was carried out noting two measurements in the supine, in sitting and in standing position, spaced apart from 1–2 minutes. For the current study mean values in sitting position were considered. BP measurements were assessed by trained personnel using a dedicated, upper arm, electronic machine (Afib screen, Microlife, Italy).

### D. Anthropometric parameters

The weight classes were defined by BMI [weight(kg)/height (m)^2^]. In adults, overweight is identified by a BMI of 25–29.9 kg/m2, and obesity by a BMI ≥30 kg/m^2^.

### E. Biochemical parameters

For each patient, the following laboratory tests were evaluated: fasting glucose, total cholesterol, LDL, HDL, triglycerides, blood urea nitrogen (BUN), serum creatinine and creatinine clearance (calculated with MDRD or Cockroft formula). Fasting blood glucose greater than 126 mg/dl was used for screening for diabetes.

### F. Follow-up with computerized medical records

The patient population was included in a central database that uses Wincare software (TSD-Projects, Milan, Italy), which contains separate electronic sheets for medical history, physical examination, laboratory tests, electrocardiogram, cardiac ultrasounds, other imaging tests and ambulatory blood pressure monitoring. The data was updated at each follow-up visit with a revaluation deadline set at three months. The data of each patient are stored on the hospital server and protected by a firewall system with password access.

### G. Echocardiography

All patients were subjected to one-dimensional echocardiography (M-mode), two-dimensional (B-mode) and Doppler function via the 5–1MHz probe (E9, GE Healthcare).

### H. Statistical analysis

Categorical data are presented as percent while continuous data are indicated as means ± standard error. The quantitative analysis was performed using T-test for unpaired data or ANOVA as appropriate, while the qualitative analysis was performed using non-parametric tests (χ^2^ test). A value of p-value <0.05 was considered statistically significant. All data were analyzed using Prism 6.0 (GraphPad Software, Inc., San Diego, CA).

## III. RESULTS

### A. Patient disposition and baseline characteristics

We considered 39 patients with hypertension, aged between 35 and 70 years, with a recent diagnosis of hypertension and initiated to treatment with Perindopril/Amlodipine. Patients were randomized to fixed-dose (Fixed, n=26) or to free dose (Free, n=13) combination therapy.

The anthropometric parameters of the two groups are similar (Table l), and the two groups did not differ as regards to biochemical and metabolic parameters, kidney damage (Table 2), and cardiac damage (left ventricular mass index: Fixed: 139.80±8.48 vs Free: 136.14±9.28g/m^2^, n.s.). Patients were not diabetic, while 13 Fixed vs 9 Free patients take antilypidemic therapy. Basal values of BP were overlapping between the two groups, with an average value of SBP and DBP, respectively, of 155.00±4.34 mmHg and 92.27±2.94 mmHg in the fixed-dose group, and of 151.54±5.75 mmHg and 85.85±2.83 mmHg in free dose group. Perindopril and Amlodipine doses did not significantly differ between the two groups, although patients with a fixed dose received a lower albeit not significant dosage of Perindopril (Fixed= Perindopril 8.85±0.44 mg, Amlodipine 7.12±0.50 mg vs Free= Perindopril 10.50±1.68 mg, Amlodipine 7.50±0.80 mg; n.s.).

### B. Comparison of BP after treatment

**Figure 1:**
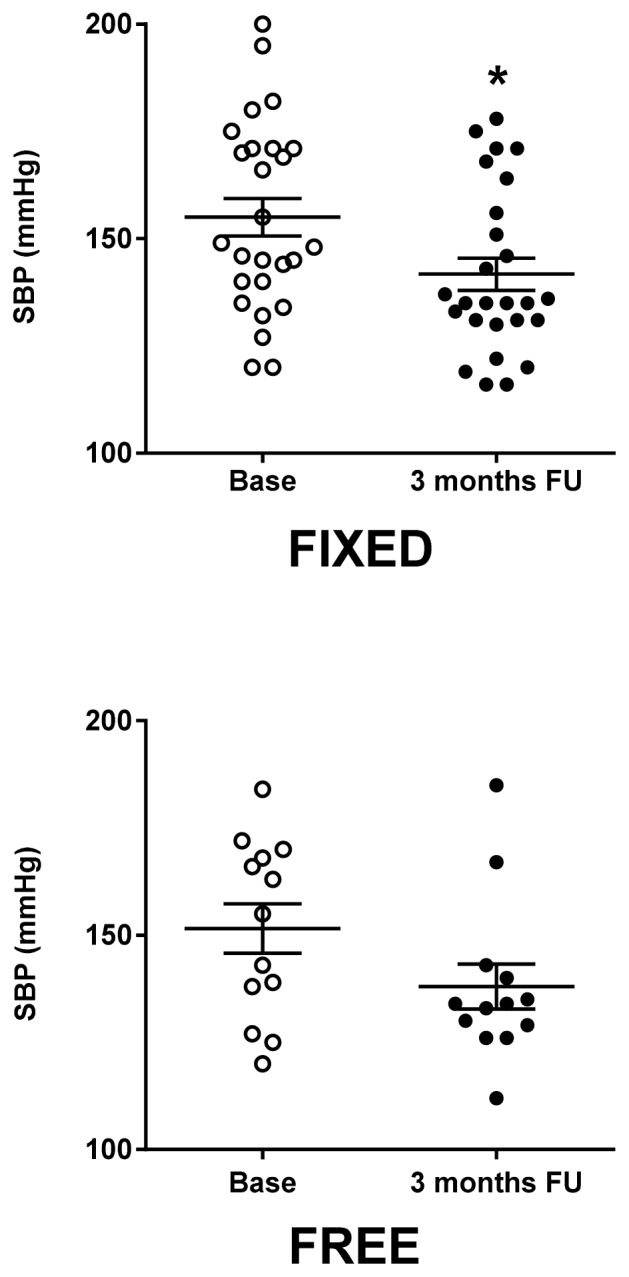
Effects of once-daily fixed dose vs free dose combination therapy on systolic blood pressure (SBP). Fixed group (Left graph) showed a statistically significant reduction of SBP, while the reduction of Free group (Right graph) was not significant. Statistic significance was assessed by T-test (*= p<0.05; FU= follow-up).

Three months after the beginning of the treatment, the percent of patients that achieved good control (<140/90 mmHg) of BP was similar between the fixed and free combination groups, both for SBP (Fixed: 61.54 vs Free: 69.23%; n.s.) and DPB (Fixed: 80.77 vs Free: 84.62%; n.s.). Furthermore, the reduction of SBP was observed in both groups (Figure 1).

**Figure 2:**
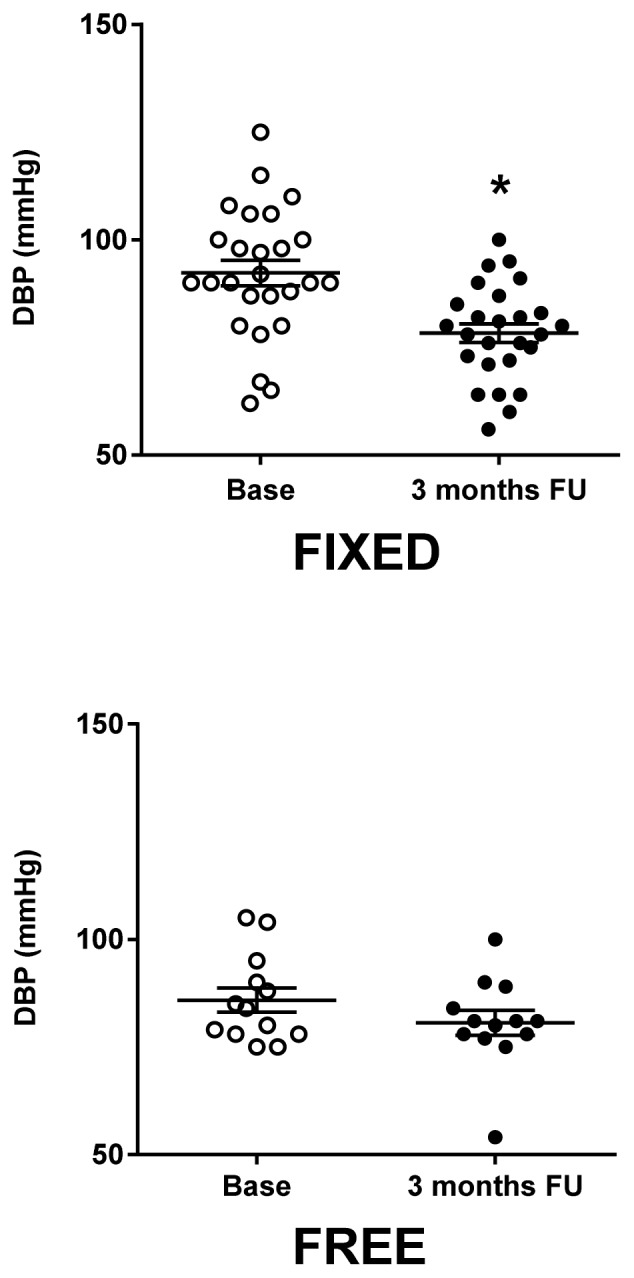
Effects of once-daily fixed dose vs free dose combination therapy on diastolic blood pressure (DBP). Fixed group (Left graph) showed a statistically significant reduction of DBP, while the reduction of Free group (Right graph) was not significant. Statistic significance was assessed by T-test (*= p<0.05; FU= follow-up).

**Figure 3:**
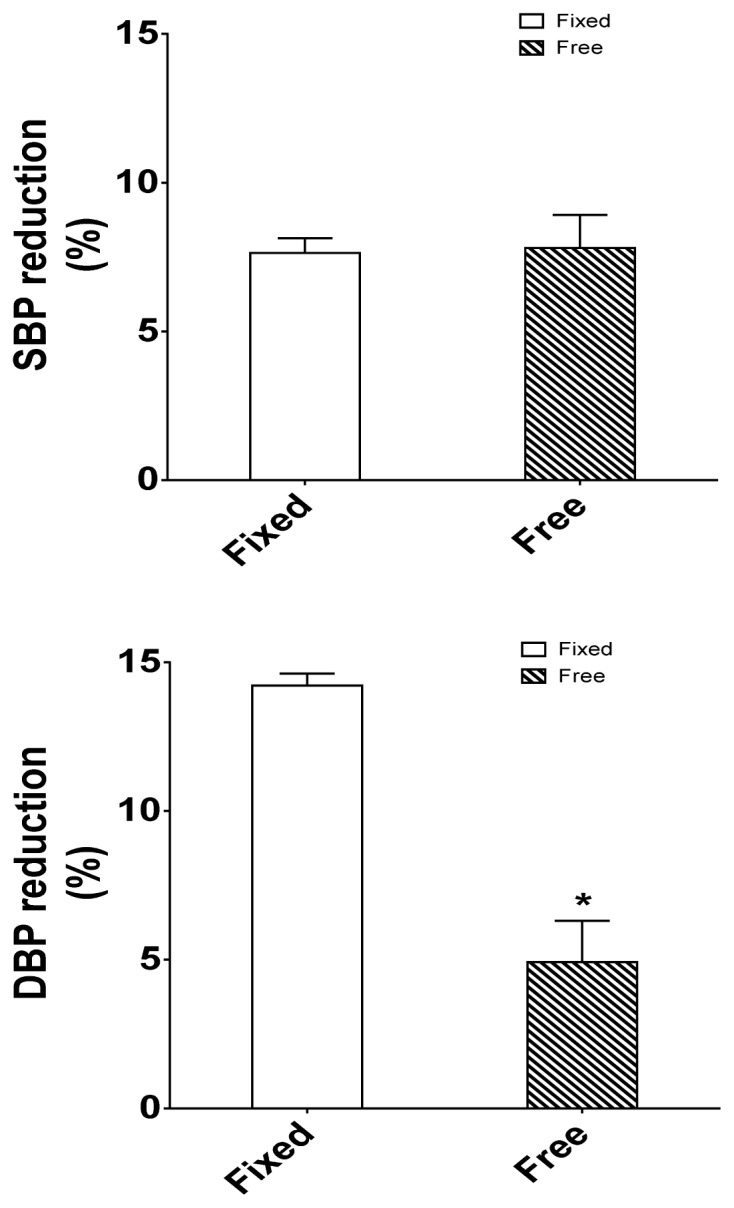
Differences between fixed dose vs free dose combination therapy on blood pressure reduction. The reduction in systolic blood pressure (SBP) (Left graph) showed no major differences between the two groups, while the reduction of diastolic blood pressure (DBP) (Right graph) was significantly higher. Statistic significance was assessed by T-test (*= p<0.05).

Similarly, DBP values were improved (Fixed: 78.35±2.17 mmHg; Free: 80.62±2.90 mmHg) (Figure 2). To evaluate the amplitude of effectiveness of treatment, we assessed the delta of BP, that is the ratio between the values of BP at the end of the treatment over those registered at baseline. The reduction in systolic blood pressure showed no major differences between the two groups (Fixed: 7.64±2.49 vs Free: 7.81±4.00%, n.s.), while the reduction in diastolic blood pressure (Figure 2) was significantly larger in the Fixed group (Figure 3).

## IV. DISCUSSION

The results of this study demonstrate for the first time that the fixed combination therapy of ACEi/CCB exerts better antihypertensive response than the free dose combination therapy of the same two drugs. Our results indicate that this effect is particularly true for diastolic blood pressure, more than for SBP. We did not investigate the underlying mechanism, which can be related to the pharmacokinetics and pharmacodynamics involved in the administration of the drugs and how they are affected by administration modality.

In this regard, it was reported that the pharmacokinetic profile of an ACE inhibitor/ARB is not affected by co-administration of a calcium channel blocker, showing that the peak occurs after less than four hours, during both the monotherapy and the combination with a calcium antagonist^19^. On the contrary, the peak plasma concentration in chronic treatment with calcium antagonist was reached after 8 hours when administered alone or in combination with olmesartan^19^. This observation rules out any significant pharmacokinetic interaction between ACE-inhibitor/CCB which could lead to the reduction of the antihypertensive cumulative or adverse side effects^19^ and suggests that the pharmacokinetics interferes less with the treatment compared to pharmacodynamic.

Another possible mechanism that can be considered to explain the result is compliance and adherence to therapy. In our study, we assessed the adherence to therapy by interviews at the time of visit. All patients referred to be adherent to treatment, a statement that in our study design could not be verified. A poor adherence to treatment or a delay in the assumption of the second pill can both explain the difference in terms of BP reduction observed between our two populations.

Treatment compliance, adherence, and persistence are key factors to achieve and maintain BP control, reduce mortality and improve the quality of life^20^, and reduce health care costs^21^. In fact, poor adherence not only implies an increase in cardiovascular mortality but also the increase of social and individual costs. Obstacles to adherence to treatment include tolerability, the number of drugs and the complexity of the drug regimen^22^, ^23^. In fact, increasing the dose of a single antihypertensive drug in an attempt to achieve an adequate pharmacological response can lead to an increase in side effects, resulting in the reduction of compliance and failure to achieve blood pressure goals. The combination therapy using two or more drugs with different and complementary action mechanisms offers the opportunity to improve control of blood pressure and, using the lowest drug doses, reduce the risk of side effects^24^. Our results indicate that, indeed, fixed therapy obtains even larger BP reduction using tendentially lower doses of the drugs.

Recent guidelines recommend the use of combination therapy as an alternative to monotherapy as initial treatment, in particular in patients at high cardiovascular risk^25^. In this regard, it was observed a significant increase in compliance and persistence to combination therapy administered once, in line with the results of a recent meta-analysis that evaluated the use of combination therapies administered once for various chronic diseases, such as diabetes mellitus, hypertension, and HIV^17^. Finally, it is observed a reduction of 20% of adverse events associated with the use of combination therapy administered once^26^, ^27^. Adverse events associated with the use of two-drug combinations were lower than those of the two drugs given separately^28^. However, the use of ACE/ARB+ CCB administered once in the treatment of hypertension is still not widespread, although hypertensive patients often have a complex treatment regimen, associated with a poor compliance^29^. For what concern social costs, most of the times the cost of the most used combination for hypertension administered once^30^ are lower than the individual drugs. In addition, abundant data demonstrate a clear inverse relationship between increased compliance with treatment and health care costs^31^.

Our results show an incredibly high level of BP control in naïve patients. Indeed, it is estimated that less than % of hypertensive patients in active treatment achieves BP control. Our results show BP control in almost 70% of patients after 3 months of treatment. Contributing to this result are the short duration of follow-up (3 months) and the use of a telematic follow-up, that has proven to be able to improve the compliance to treatment in previous studies^32^. The use of both a management strategy of the hypertension therapy and an appropriate drug regimen might help to achieve in real life a rapid control of BP. This is an important advantage, as it produces a more effective control of the cardiovascular risk, especially in hypertensive patients with multiple conditions, (diabetes, cerebrovascular, cardiovascular or renal disease), that more frequently require multiple therapies. According to the ESH/ESC 2013 guidelines, combination therapy must be considered the initial treatment step in these patients. Our result justifies the use of fixed dose to achieve a larger control of BP.

In conclusion, although both fixed and free dose ACEi/CCB combination therapy are effective in reducing BP, the fixed doses in a single tablet has a particular advantage in the reduction of BP. This advantage must be taken into account in the choice of the drug, especially in the presence of high cardiovascular risk.

## Figures and Tables

**Table 1:** Anthropometric parameters of the study participants. Data are presented as means±standard error unless otherwise indicated. (n.s.= not significant).

Variable	FIXED DOSE (n=26)	FREE DOSE (n=13)	p-value
AGE (years)	61.79±2.28	64.23 ±2.45	n.s.
WOMEN (%)	31	31	n.s.
BMI (Kg/m^2^)	29.68±1.39	29.73±1.10	n.s.
HEIGHT (cm)	167.12±2.09	165.77±2.00	n.s.
WEIGHT (Kg)	82.38±3.59	81.85±3.86	n.s.

**Table 2:** Biochemical parameters of the study participants. Data are presented as means±standard error. (n.s.= not significant).

Variable	FIXED DOSE (n=26)	FREE DOSE (n=13)	p-value
GLYCEMIA (mg/dl)	105±18	104±10	n.s.
TOTAL CHOLESTEROL (mg/dl)	168±32	182±48	n.s.
LDL(mg/dl)	91±28	106.3±45	n.s.
HDL(mg/dl)	52±18	46±10	n.s.
TRIGLYCERIDES (mg/dl)	119±40	131±44	n.s.
BUN (mg/dl)	41±7	44±9	n.s.
CREATININE (mg/dl)	0.87±0.17	0.88±0.17	n.s.
CREATININE CL (ml/min)	100±46	92±21	n.s.
